# Immune cell C/EBPβ deficiency is associated with hepatic mononuclear defects and spontaneous hepatitis but not steatohepatitis induced liver fibrosis

**DOI:** 10.1002/iid3.728

**Published:** 2022-10-27

**Authors:** Itay Moshkovits, Ayelet Kaminitz, Debby Reuveni, Metsada Pasmanik‐Chor, Eli Brazowski, Alexander Mildner, Achim Leutz, Ehud Zigmond

**Affiliations:** ^1^ Research Center for Digestive Diseases Tel Aviv Sourasky Medical Center Tel Aviv Israel; ^2^ Sackler Faculty of Medicine Tel Aviv University Tel Aviv Israel; ^3^ Center for Autoimmune Liver Diseases Tel Aviv Sourasky Medical Center Tel Aviv Israel; ^4^ Bioinformatics Unit, George‐S. Wise Faculty of Life Science Tel Aviv University Tel Aviv Israel; ^5^ Department of Pathology Tel Aviv Sourasky Medical Center Tel Aviv Israel; ^6^ Max Delbrück Center for Molecular Medicine Berlin Germany

**Keywords:** cells, molecules, monocytes/macrophages, transcription factors

## Abstract

**Background:**

CCAAT/enhancer‐binding protein β (C/EBPβ) is a transcription factor known to be involved in macrophage differentiation and function, steatohepatitis and liver fibrosis.

**Methods:**

Immune restricted C/EBPβ deficient and control mice were investigated in steady‐state and in the CDA‐HFD steatohepatitis model. Mice were assessed for weight change, liver biochemical profile, histology and hepatic phagocytes composition.

**Results:**

Flow cytometry analysis of hepatic nonparenchymal cells revealed reduced numbers of hepatic monocytes and Kupffer cells and an increase in hepatic MHC class II positive myeloid cells in immune cells restricted C/EBPβ deficient mice. Immune‐restricted C/EBPβ deficiency resulted in decreased weight gain and appearance of mild spontaneous liver inflammation. Nevertheless, In the CDA‐HFD steatohepatitis model, immune restricted C/EBPβ deficient and proficient mice exhibit similar grade of hepatic steatosis, liver enzymes levels and fibrosis stage.

**Conclusions:**

Immune‐restricted C/EBPβ deficiency leads to significant alteration in hepatic mononuclear phagocytes composition associated with spontaneous mild hepatitis. Steatohepatitis associated fibrosis is not dependent on C/EBPβ expression by immune cells.

## Graphical abstract

Investigating the liver of immune restricted C/EBPβ‐deficient mice revealed major alteration in the composition of the intra‐hepatic myeloid cells pool associated with mild spontaneous hepatitis. Yet, immune cell restricted C/EBPβ‐deficiency did not confer protection from hepatic steatosis, inflammation and fibrosis, that are suggested to be influenced by C/EBPβ expression in nonimmune cells.

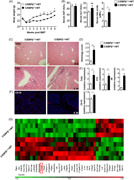

## 1 INTRODUCTION

The CCAAT/enhancer‐binding protein (C/EBP) proteins are a family of basic region leucine zipper transcription factors that includes six members.^1^ These proteins are composed of basic region and leucine zipper sequences (bZip) that mediate DNA binding and dimerization and a N‐terminal effector domain. The four “activating” C/EBPs (α, β, δ, ε) can bind to palindromic DNA sites as homo‐ or heterodimers and have been described in gene regulation in many physiological and pathological conditions, including innate and adaptive immunity, inflammation, hematopoiesis, adipogenesis, cell cycle, cancer and tissue development.^2^, ^3^

One member of the C/EBPs family is C/EBPβ. Protein expression of the single exon C/EBPβ transcript is regulated by alternative translation initiation. The C/EBPβ transcript encodes products known as liver activating proteins, LAP* and LAP, and liver inhibitory protein (LIP) which retains the bZip, lacks the major N‐terminal transactivation domain and can dominantly inhibit the activating C/EBP isoform.^4^ Genetic studies evaluating C/EBPβ suggested context specific and cell type specific functions and contribution to growth arrest, induction of senescence, but also to cell proliferation.^2^

Within the hematopoietic system, C/EBPβ is involved in regulation of the development and function of macrophages.^5^ Enforced expression of C/EBPβ in differentiated B cells leads to their reprogramming into macrophages.^6^ Moreover, macrophages deficient for C/EBPβ exhibit impairment of bactericidal and antitumor cytotoxic activity.^7^ C/EBPβ was found to be important for the development of two types of tissue resident macrophages, large peritoneal macrophages and alveolar macrophages, while macrophage development in steady state conditions in other organs including the spleen, skin and kidney, seemed to be normal.^8^ Recently, it has been shown that the generation of Ly6C^low^ cells involves the induction of C/EBPβ and that C/EBPβ‐deficient mice lack Ly6C^low^ monocytes.^9^ Moreover, another recently published study revealed that C/EBPβ is essential for the development of a specific type of cells, resembling Ly6C^low^ monocytes by their surface markers expression, suggested to be involved in fibrosis formation in various organs.^10^

In the liver, C/EBPβ was shown to be required for normal hepatocyte proliferation in a murine model of partial hepatectomy.^11^, ^12^ Hepatic C/EBPβ messenger RNA  (mRNA) levels are upregulated in acute‐phase response to inflammatory stimuli by lipopolysacharide and various proinflammatory cytokines.^13^, ^14^, ^15^ Notably, C/EBPβ‐deficient mice are protected from lipid accumulation and hepatic inflammation in a model of diet‐induced steatohepatitis,^16^ and protected from the development of liver fibrosis in a murine model of chronic exposure to the hepato‐toxin carbon tetrachloride (CCl4).^17^ However, it is unknown whether C/EBPβ specific expression by immune cells rather than structural cells in the liver is required for hepatic homoeostasis.

Herein we aim to investigate the role of immune system specific C/EBPβ expression in the liver at steady state and following a diet‐induced steatohepatitis model.

## 2 MATERIALS AND METHODS

### 2.1 Animals

Female C57BL/6 wild type (WT) mice were purchased from Envigo (Ness‐Ziona, Israel). CEBP/β^+/+^ and CEBP/β^−/−^ mice were kindly provided by Prof. Achim Leutz, the Max‐Delbrueck‐Center for Molecular Medicine (MDC), Berlin, Germany.

Mice were maintained in the animal facility of the Tel‐Aviv Sourasky Medical Center and had unrestricted access to food and water, were housed in temperature and humidity‐controlled rooms and were kept on a 12 h light/dark cycle. Use of animals was in accordance with the National Institutes of Health policy on the care and use of laboratory animals and was approved by the Tel‐Aviv Sourasky Medical Center Animal Use and Care Committee.

### 2.2 Bone marrow (BM) irradiated chimeras

Eight‐week‐old recipient male WT C57BL/6 mice were subjected to whole‐body irradiation (950 rad) using a TrueBeam linear accelerator (Varian Medical Systems). The following day, BM cells were harvested from the hind limbs (tibia and femur) of the appropriate donor mice (CEBP/β^+/+^ and CEBP/β^−/^^−^) and 2 × 10^6^ cells were injected i.v. into tail vein of the irradiated recipients. Mice were allowed to reconstitute their BM cells for 8 weeks before conducting experiments.

### 2.3 CDA‐HFD steatohepatitis model

Chimeric mice were fed for 8 weeks with the l‐amino acid defined diet with 60 kcal% fat with 0.1% methionine and no choline added (CDA‐HFD (A06071302), Research Diets). A normal chow diet (provided by the Animal Facility at the Tel‐Aviv Sourasky Medical Center) was used as a control diet.^18^

### 2.4 Isolation of hepatic nonparenchymal cells and flow cytometry

Isolation of hepatic nonparenchymal cells was performed as previously described.^19^ Cell preparations were incubated with monoclonal antibody 2·4G2 for FcR blocking (BioLegend) and then exposed at 4°C to a combination of the following antibodies: anti‐mouse CD45 (clone 30‐F11), anti‐mouse/human CD11b (clone M1/70), anti‐mouse Ly6C (clone HK1.4), anti‐mouse MHCII (clone M5/114.15.2), anti‐mouse ceacam1 (clone CC1) all were purchased from BioLegend, San Diego, CA. Anti‐mouse F4/80 (clone REA126) and anti‐mouse Tim4 (clone REA999) was purchased from Miltenyi Biotec. Cells were analyzed with BD FACSCanto™ II (BD Bioscience). Flow cytometry analysis was performed using FlowJo software.

### 2.5 Histopathology

Livers from euthanized mice were fixed in Formaldehyde 4% buffered (pH 7.2), embedded in paraffin, cut into 4‐μm sections, deparaffinized and stained with hematoxylin and eosin. Disease severity was evaluated by an experience pathologist (E.B.) who was blinded to treatment allocation. Scoring was done as previously described^20^ considering the following parameters: hepatocellular steatosis, inflammatory cell infiltration, and hepatocellular ballooning.

### 2.6 Immunofluorescence staining

Liver lobes were fixed in 4% paraformaldehyde overnight in 4°C. Then, lobes were placed in 30% sucrose in phosphate‐buffered saline (PBS) until tissue sinks (6–12 h) and embedded in OCT.

Immunofluorescent staining was performed on 13 µm frozen liver sections. Slides were incubated in cold acetone for 7 min and dried at room temperature. Following washing, slides were blocked with 1% bovine serum albumin for 1 h at room temperature. Samples were stained overnight at 4°C with anti‐CK19 (Abcam) to identify bile ducts. Slides were washed 3 times for 5 min in PBS^−/−^ and stained with secondary antibody (Donkey Anti‐Rabbit IgG H&L, 1:1000, Alexa Fluor 647; Abcam) for 1 h at room temperature.

Eventually the slides were cover slipped with Fluorescent Mounting Medium with 4,6‐diamidino‐2‐phenylindole (DAPI; GBI Labs). Images were taken with ZEISS Confocal Microscope (MicroImaging GmbH, ZEISS). Processing was performed with ZEN 2010 software.

### 2.7 Fibrosis evaluation

Picro‐Sirius red staining was performed using a commercially available kit (ab150681; Abcam) according to the manufacturer's protocol. Tissue collagen accumulation was quantitatively assessed by a hydroxyproline accumulation assay (MAK008; Sigma‐Aldrich) according to the manufacturer's instructions.

### 2.8 Quantitative real‐time PCR

RNA from livers was isolated with the RNeasy Micro kit (QIAGEN) and reverse‐transcribed with a High‐Capacity complementary DNA (cDNA) Reversed transcription kit (Applied Biosystems) according to the manufacturer's instructions. PCRs were performed with the SYBER green PCR Master Mix (Applied Biosystems). Quantification was done with Step One software (V2.2). The genes of interest; Collagen1, transforming growth factor β (TGF)‐β, α‐smooth muscle actin (αSMA), tumor necrosis factor α (TNF‐α), Ck19, interleukins (IL)‐10 and IL‐6 were compared with ribosomal protein large P0 (RPLP0) housekeeping gene. Primer sequences (forward and reverse, respectively) were:

RPLPO, 5′‐TCCAGCAGGTGTTTGACAAC‐3′ and 5′‐CCATCTGCAGACACACACT‐3′;
Coll‐I, 5′‐GAGAGCATGACCGATGGATT‐3′ and 5′‐CCTTCTTGAGGTTGCCAGTC‐3′;
TGF‐β, 5′‐ATTCAGCGCTCACTGCTCTT‐3′ and 5′‐GTTGGTATCCAGGGCTCTCC‐3′;
αSMA, 5′‐TGATCACCATTGGAAACGAA‐3′ and 5′‐CCCCTGACAGGACGTTGTTA‐3′;
TNF‐α, 5′‐CGAGTGACAAGCCTGTAGCC‐3′ and 5′‐CCTTGTCCCTTGAAGAGAACC‐3′;
IL‐10, 5′‐TGCTATGCTGCCTGCTCTTA‐3′ and 5′‐ATGTTGTCCAGCTGGTCCTT‐3′;
IL‐6, 5′‐ACAGTGTGGGAAGCAAGTCC‐3′ and 5′‐TCGGTATCGAAGCTGGAACT‐3′;
CK19, 5′‐TGATCGTCTCGCCTCCTACT‐3′ and 5′‐CAAGGCGTGTTCTGTCTCAA‐3′

### 2.9 RNAseq libraries preparation

Liver Ly6c+ cells were sorted into 40 μl of lysis/binding buffer (Life Technologies) and stored at 80°C. mRNA was captured with Dynabeads oligo(dT) (Life Technologies) according to manufacturer's guidelines. A bulk variation of MARSseq^21^ was used to prepare libraries for RNA‐seq. Briefly, RNA was reversed transcribed with MARSseq barcoded RT primer in a 10 μl volume with the Affinity Script kit (Agilent). Reverse transcription was analyzed by qRT‐PCR and samples with a similar computerized tomography were pooled (up to eight samples per pool). Each pool was treated with Exonuclease I (NEB) for 30 min at 37°C and subsequently cleaned by 1.2× volumes of SPRI beads (Beckman Coulter). Subsequently, the cDNA was converted to double‐stranded DNA with a second strand synthesis kit (NEB) in a 20 ml reaction, incubating for 2 h at 16°C. The product was purified with 1.4× volumes of SPRI beads, eluted in 8 μl and in vitro transcribed (with the beads) at 37°C overnight for linear amplification using the T7 High Yield RNA polymerase IVT kit (NEB). Following IVT, the DNA template was removed with Turbo DNase I (Ambion) 15 min at 37°C and the amplified RNA (aRNA) purified with 1.2 volumes of SPRI beads. The aRNA was fragmented by incubating 3 min at 70°C in Zn2+ RNA fragmentation reagents (Ambion) and purified with 2× volumes of SPRI beads. The aRNA was ligated to the MARS‐seq ligation adaptor with T4 RNA Ligase I (NEB). The reaction was incubated at 22°C for 2 h. After 1.5× SPRI cleanup, the ligated product was reverse‐transcribed using Affinity Script RT enzyme (Agilent) and a primer complementary to the ligated adaptor. The reaction was incubated for 2 min at 42°C, 45 min at 50°C, and 5 min at 85°C. The cDNA was purified with 1.5× volumes of SPRI beads. The library was completed and amplified through a nested PCR reaction with 0.5 mM of P5_Rd1 and P7_Rd2 primers and PCR ready mix (Kappa Biosystems). The amplified pooled library was purified with 0.7× volumes of SPRI beads to remove primer leftovers. Library concentration was measured with a Qubit fluorometer (Life Technologies) and mean molecule size was determined with a 2200 TapeStation instrument. RNaseq libraries were sequenced using the Illumina NextSeq. 500.

### 2.10 RNAseq analysis

Data analysis was performed by using the UTAP transcriptome analysis pipeline. Raw reads were trimmed using cutadapt with the parameters: ‐a AGATCGGAAGAGCACACGTCTGAACTCCAGTCAC ‐a “A–times 2 ‐u 3 ‐u −3 ‐q 20 ‐m 25). Reads were mapped to the genome (mm10, Gencode annotation version 10.0) using STAR (v2.4.2a) with the parameters –alignEndsType EndToEnd, –outFilterMismatchNoverLmax 0.05, –twopassMode Basic, –alignSoftClipAtReferenceEnds No. The pipeline quantifies the 3′ of Gencode annotated genes (The 3′ region contains 1000 bases upstream of the 3′ end and 100 bases downstream). UMI counting was done after marking duplicates (in‐house script) using HTSeq‐count in union mode. Only reads with unique mapping were considered for further analysis, and genes having minimum 5 reads in at least one sample were considered. Gene expression levels were calculated and normalized using DESeq. 2 with the parameters: betaPrior = True, cooksCutoff = FALSE, independentFiltering = FALSE. Batch correction was done using the sva (3.26.0) R when batch adjustments were required. Raw *p* values were adjusted for multiple testing, using the procedure of Benjamini and Hochberg. Differentially expressed genes were selected with *p*‐value of false discovery rate < 0.05 and fold‐change difference = 2. Visualization of gene expression heatmaps was done using Partek Genomics Suite (https://www.partek.com/partek-genomics-suite/), using log normalized values. Hierarchical clustering was applied, using the Pearson's Dissimilarity algorithm (Ward's method). For Gene Ontology term analysis, DAVID function enrichment were used.

### 2.11 Statistical design and analysis

The results are presented as mean ± *SEM*. Statistical significance was assessed using a two‐tailed Student *t* test; *p* < .05 were considered as significant. In all experiments no criteria for inclusion or exclusion animals or other experimental units were used.

No randomization was used to allocate experimental units to control or treatment groups and potential confounders such as order of treatments and measurements were not systematically controlled.

## 3 RESULTS

### 3.1 Immunophenotyping of hepatic phagocytes in C/EBPβ chimera mice

C/EBPβ plays an important role in the development and differentiation of myeloid cells.^8^, ^9^, ^13^ To test whether C/EBPβ specific expression by immune cells rather than structural cells in the liver is required for hepatic homeostasis, we have generated bone marrow chimeric mice by transplanting C/EBPβ^−/−^ or C/EBPβ^+/+^ bone marrow into irradiated‐WT mice recipients.

**Figure 1:**
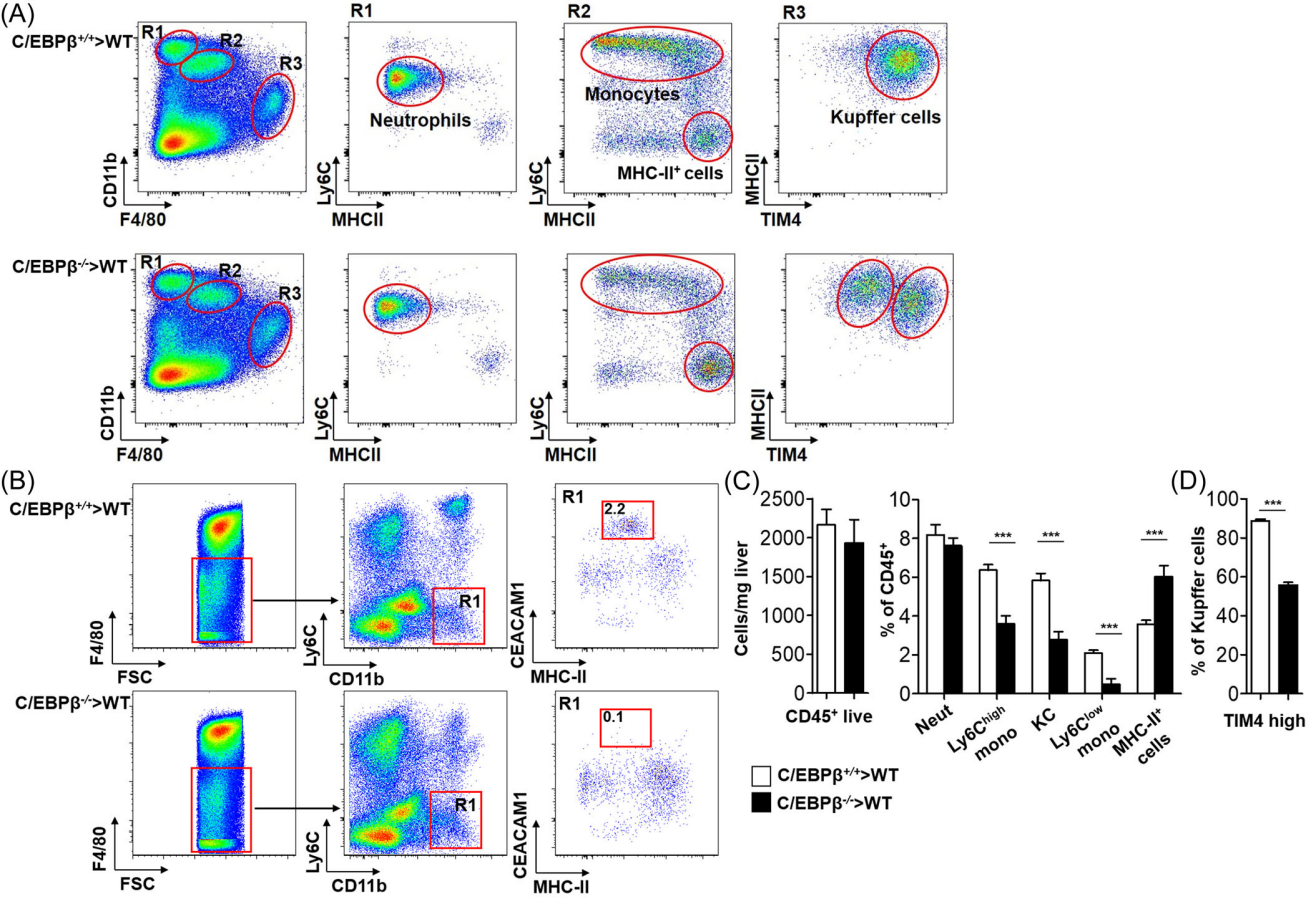
Altered hepatic myeloid cell populations in immune cell restricted C/EBPβ deficient mice. Single cell suspensions of hepatic cells were obtained 8 weeks after bone marrow transplantation of C/EBPβ^−/−^ or C/EBPβ^+/+^ bone marrow into irradiated wild type recipients mice. Representative flow cytometry dot plots of singlets, CD45+ live (DAPI negative) cells including gating strategy for neutrophils, Ly6C^+^ monocytes, MHC II positive myeloid cells and Kupffer cells are depicted (A). Gating and representative dot plots of hepatic Ly6C^−^ monocytes in indicated mice (B). Quantification of myeloid cell counts in the livers of indicated mice (C). Precent of TIM4^+^ Kupffer cells in indicated mice (D). Data is a representative experiment of *n* = 2 (9 mice per experimental group). *p < .05, **p < .01, ***p < .001. C/EBPβ, CCAAT/enhancer‐binding protein β

Following bone marrow transplantation and recovery interval, several phagocyte populations were identified in the liver (Figure 1A), including neutrophils (gated as CD11b^high^/F4‐80^neg^/Ly6C^dim^), Ly6C^high^ monocytes (gated as CD11b^pos^/F4‐80^low^/Ly6C^high^), MHC II positive myeloid cells (gated as CD11b^pos^/F4/80^low^/Ly6C^neg^/MHCII^high^), Kupffer cells (gated as CD11b^dim^/F4‐80^high^/MHCII^pos^) and Ly6C^low^ monocytes (gated as F4/80^neg^/CD11b^pos^/Ly6C^neg^/CEACAM1^pos^; Figure 1B).

**Figure 2:**
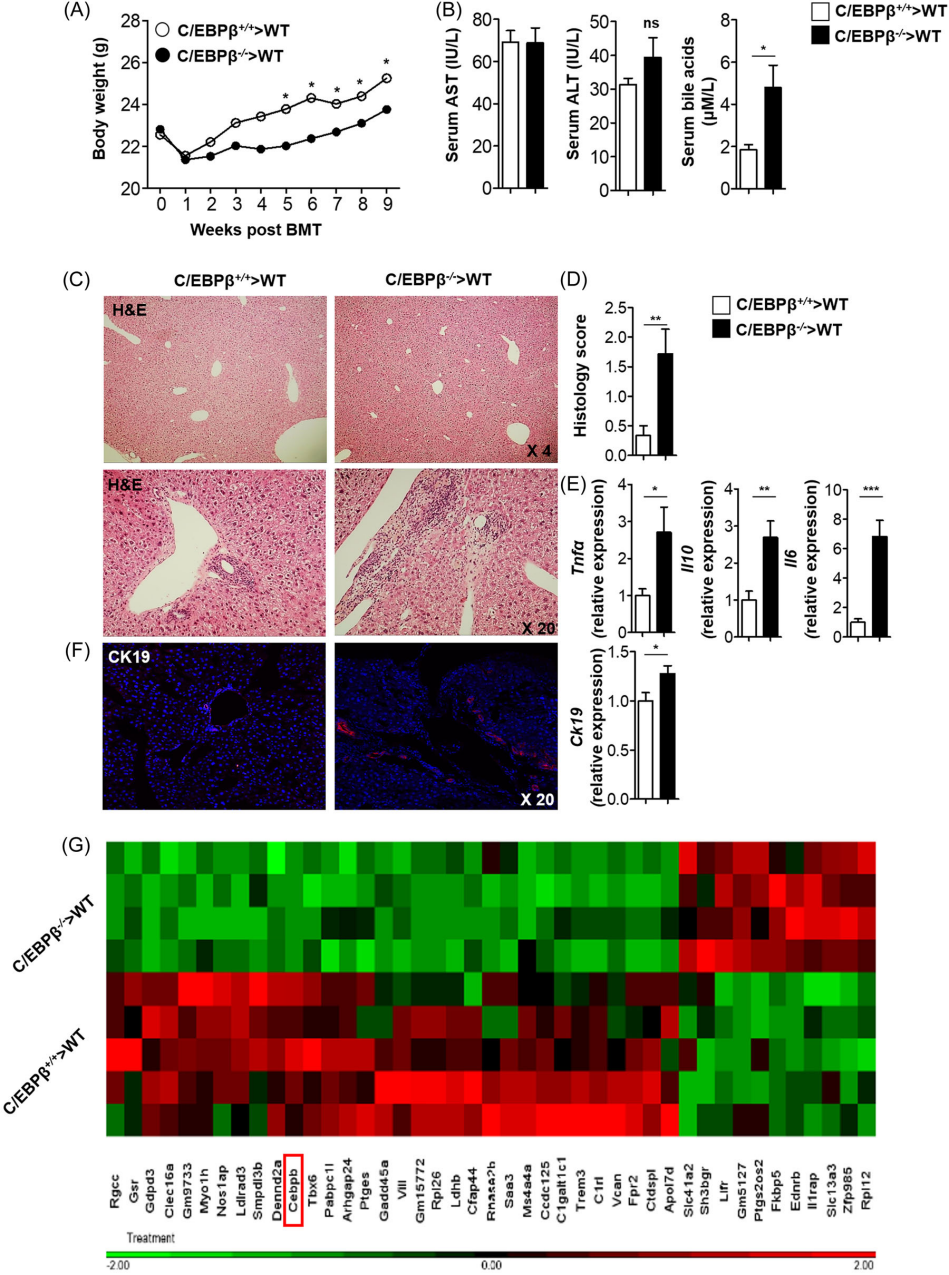
Immune cell restricted C/EBPβ deficient mice exhibit weight gain retardation and mild spontaneous hepatitis Body weight of C/EBPβ^−/−^ or C/EBPβ^+/+^ chimeric mice following irradiation and BM transfer (A). Serum AST, ALT, and bile acids levels measured 8 weeks following BM transfer (B). Representative Hematoxylin and Eosin staining of liver sections 8 weeks following BM transfer (C). Liver histology severity score (D). Hepatic cytokines mRNA 8 weeks following BM transfer (E). Liver immunofluorescence staining for CK19 and hepatic *Ck19* mRNA levels 8 weeks following BM transfer (F). RNA‐Seq analysis of differential expressed genes in Ly6C^high^ monocytes sorted from the livers of C/EBPβ^−/−^ or C/EBPβ^+/+^ chimeric mice following irradiation and BM transfer (G). For (A) and (B) data is pooled from *n* = 3 (7–10 mice per group in each experiment). For (C), (D), (E), and (F) data is a representative experiment of *n* = 3 (9 mice per experimental group). For (G) data is of *n* = 4–5 mice in each group. *p < .05, **p < .01, ***p < .001. BM, bone marrow; C/EBPβ, CCAAT/enhancer‐binding protein β; mRNA, messenger RNA

Importantly, quantitative analysis of C/EBPβ mRNA expression by sorted liver Ly6C^high^ monocytes revealed a 10 times fold change expression in C/EBPβ^+/+^ control mice compared to C/EBPβ^−/−^ chimeric mice (Figure 2F), which confirms high efficacy in bone marrow grafting.

Quantitative comparison of liver phagocytes in C/EBPβ^−/−^ chimeric mice and C/EBPβ^+/+^ control mice, respectively, revealed similar counts of neutrophils, lower counts of Ly6C^high^ monocytes and Kupffer cells, and increased numbers of MHC II positive myeloid cells in the immune‐restricted C/EBPβ^−/^^−^ mice (Figure 1C). In accordance with previous publications,^9^, ^10^ liver Ly6C^low^ monocytes were almost absent in the C/EBPβ^−/−^ chimera mice (Figure 1B,C). Interestingly, while almost all Kupffer cells in the livers of C/EBPβ^+/+^ control mice express high levels of TIM4, two distinct Kupffer cells population were identified in immune‐restricted C/EBPβ^−/−^ mice with nearly 50% of Kupffer cells expressing low TIM4 levels (Figure 1A and 1D). Thus, immune restricted C/EBPβ deficiency is associated with reduced numbers of hepatic monocytes and Kupffer cells, an increase in hepatic MHC II positive myeloid cells and alteration in TIM4 expression by Kupffer cells.

### 3.2 Immune restricted C/EBPβ deficiency is associated with spontaneous mild hepatitis

To examine whether alteration in liver phagocytes populations in immune restricted C/EBPβ deficient mice is associated with liver injury, C/EBPβ^−/−^ chimeric and control mice were assessed for clinical, biochemical and histological parameters. Following bone marrow transplantation, both groups expected weight loss of 10% from their baseline weight, while C/EBPβ^−/−^ chimeric mice fail to gain weight at the same pace as control mice (Figure 2A). Serum biochemical liver profile revealed comparable levels of AST and ALT between C/EBPβ^−/−^ chimeric and control mice, while bile acids were significantly elevated in the serum of C/EBPβ^−/−^ chimeric mice (Figure 2B). Histologic assessment of the liver demonstrated periportal inflammation in the livers C/EBPβ^−/−^ chimeric mice (Figure 2C) which was quantified by histology score performed by a liver pathologist (Figure 2D). In addition, hepatic transcripts levels of IL‐6, IL‐10, and TNF‐α were all elevated in C/EBPβ^−/−^ recipients, as compared to WT recipient mice (Figure 2E). Staining for CK19 and quantification of CK19 transcripts levels revealed increased cholestatic damage in the livers of C/EBPβ^−/−^ recipients (Figure 2F). Interestingly, analysis of differentially expressed genes in Ly6C^high^ monocytes sorted from the livers of C/EBPβ^−/−^ recipients mice (Figure 2G), revealed enrichment in signaling pathways of inflammatory response, leukocyte chemotaxis, and cytokine receptor activity. Thus, immune restricted C/EBPβ deficiency is associated with reduced body weight and spontaneous mild hepatitis.

### 3.3 Steatohepatitis associated fibrosis (CDA‐HFD model) is not dependent on C/EBPβ expression by immune cells

Previous reports have demonstrated attenuated liver inflammation and fibrosis in the absence of C/EBPβ expression.^17^, ^20^ To examine whether immune restricted C/EBPβ deficiency is associated with similar results we used diet‐induced steatohepatitis model by feeding the mice with methionine/choline deficient and high fat (CDA‐HFD) diet for 8 weeks.

**Figure 3:**
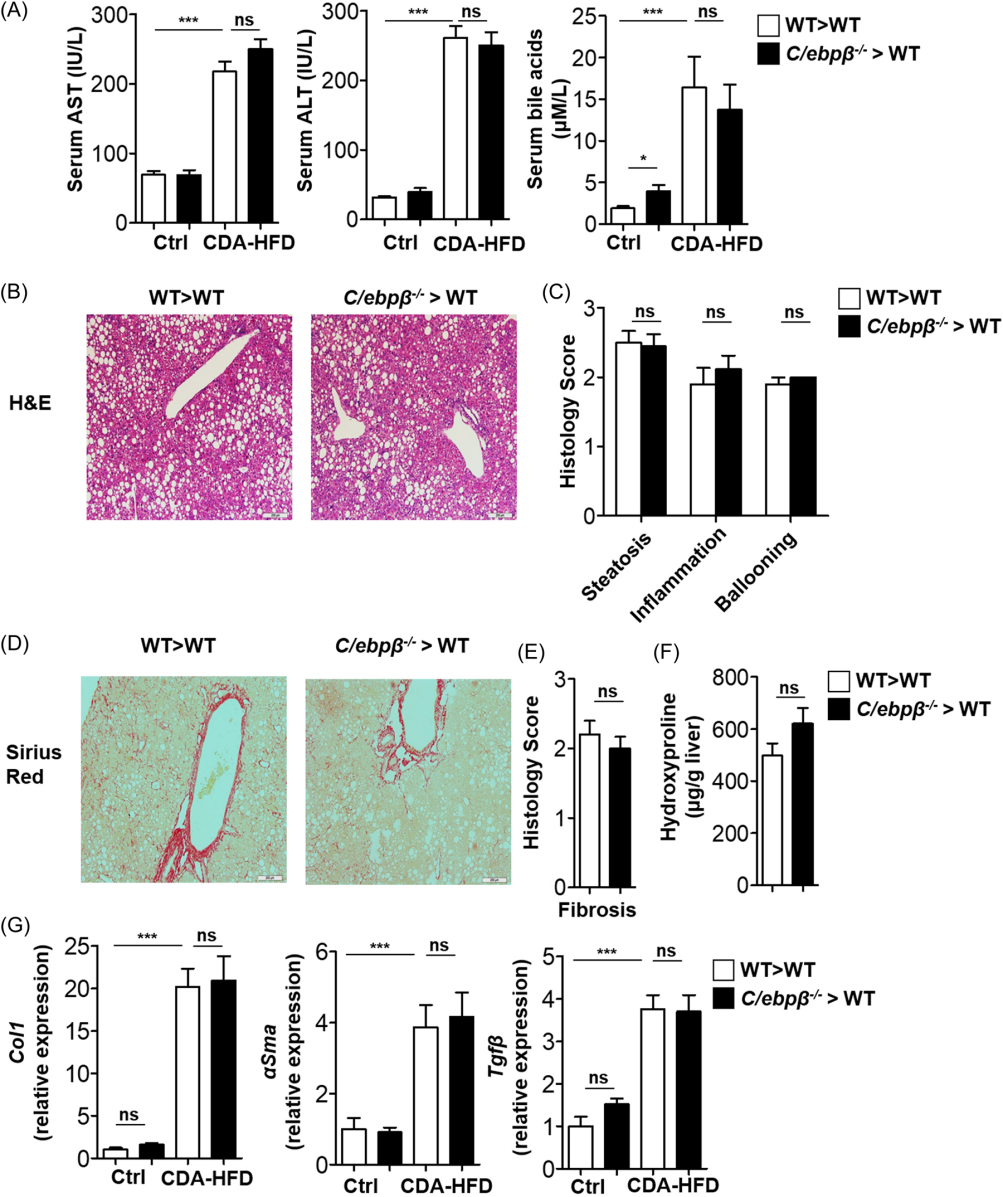
Diet‐induced steatohepatitis and liver fibrosis in immune cell restricted C/EBPβ deficient mice. C/EBPβ^−/−^ and C/EBPβ^+/+^ chimeric mice were fed with methionine/choline deficient and high fat (CDA‐HFD) diet for 8 weeks. ALT, AST, and bile acids were measured in the serum (A). Representative hematoxylin and eosin staining of liver sections (B). Histology severity score of liver steatosis, inflammation and ballooning (C). Representative Sirius red staining for collagen deposition in the liver (D). Histology severity score of liver fibrosis (E). Quantification of hydroxyproline in the liver (F). Hepatic mRNA levels following normal diet and CDA‐HFD (G). Data is pooled from *n* = 2 (9–10 mice per group in each experiment). *p < .05, **p < .01, ***p < .001. C/EBPβ, CCAAT/enhancer‐binding protein β; mRNA, messenger RNA

Following CDA‐HFD for 8 weeks, serum ALT, AST, and bile acids increased significantly but there was no significant change between C/EBPβ^−/−^ and C/EBPβ^+/+^ chimeric mice (Figure 3A). In addition, histologic analysis of the liver revealed extensive lipid accumulation in both groups and liver histology assessment failed to demonstrate any difference in steatosis, inflammation or ballooning formation between the groups (Figure 3B,C).

Similarly, following CDA‐HFD, collagen deposition in the liver, assessed by histology assessment and quantified by liver hydroxyproline levels, was not significantly different between C/EBPβ^−/−^ and C/EBPβ^+/+^ chimeric mice (Figure 3D–F). In addition, liver mRNA levels of fibrosis related genes: Collagen‐1, αSMA and TGF‐β were significantly elevated following CDA‐HFD but no differences were observed between the groups (Figure 3G). Thus, in CDA‐HFD model, steatohepatitis associated fibrosis is not dependent on C/EBPβ expression by immune cells.

## 4 DISCUSSION

The liver is composed of diverse structural cells and is also one of biggest immune organs of the body. Unraveling the role of C/EBPβ that is expressed in both, liver cells and immune cells, thus poses an experimental challenge. Herein we have taken advantage of bone marrow transfer strategy and created immune restricted deficiency of C/EBPβ expression while preserving the expression of C/EBPβ by liver structural cells.

We have demonstrated that (1) immune‐restricted deficiency of C/EBPβ is associated with altered composition of myeloid cells populations in the liver at baseline, including reduced numbers of monocytes and Kupffer cells and increased numbers of MHC II positive myeloid cells; (2) immune cell deficient C/EBPβ mice exhibit weight gain retardation and mild spontaneous hepatitis; (3) in diet‐induced steatohepatitis model, immune‐restricted C/EBPβ deficiency was not associated with reduced steatosis, inflammation or fibrosis.

Previous studies have shown that in the absence of C/EBPβ, monocyte composition in the blood and bone marrow are affected, while their precursors remain intact.^5^, ^9^ Mildner et al.^9^ have shown that C/EBPβ regulates monocyte differentiation from Ly6C^hi^ into Ly6C^low^ monocytes, while Tamura et al.^5^ have suggested that C/EBPβ is required for the survival of monocytes in the peripheral blood. Focusing on the liver myeloid cell populations for the first time and in accordance with the aforementioned reports, we now show that immune‐restricted C/EBPβ deficiency results in the absence of Ly6C^low^ monocytes in the liver and in reduced numbers of Ly6C^hi^ monocytes, but in increased numbers of myeloid MHC II positive myeloid cells. This could be explained by the tendency of C/EBPβ deficient Ly6C^hi^ monocytes for accelerated differentiation and expansion into monocyte‐derived dendritic cells.^22^

C/EBPβ deficient mice lack specific types of tissue macrophages, including large peritoneal macrophages and alveolar macrophages, while the numbers of other tissue macrophages, including the liver, where reported to be similar between C/EBPβ^−/−^ and C/EBPβ^+/+^ mice.^8^ Here, we identified lower numbers of Kupffer cells in the liver of immune‐restricted C/EBPβ^−/−^ mice. Importantly, we used similar gating strategy, identifying Kupffer cells as CD11b^int^/F4‐80^high^ cells. This difference might be explained by the contribution of C/EBPβ expression by nonimmune cells that contributes to Kupffer cells development. Alternatively, one may hypothesize that C/EBPβ is not required for the engraftment of Kupffer cells during the embryonic life but it is essential for Kupffer cells replenishment upon irradiation in a BM transfer model. T‐cell immunoglobulin and mucin domain containing 4 (Tim4) is a phosphatidylserine receptor selectively expressed on antigen presenting cells and known to be expressed by mature Kupffer cells.^23^, ^24^ Expression of Tim4 by macrophages regulates their immune activity.^25^ In contrast to WT‐Kupffer cells that express high levels of Tim4 homogenously, C/EBPβ‐deficient Kupffer cells were found to be composed of Tim4‐positive and negative populations. This could potentially be attributed to the disturbance in Kupffer cell maturation or, alternatively, decreased survival with influx of monocyte‐derived KCs that did not accomplish maturation.

Immune restricted C/EBPβ deficient mice display weight gain retardation and spontaneous mild hepatitis. A possible explanation for this phenotype could be the low numbers of KC, which act as regulatory cells and the high prevalence of myeloid DC with antigen presentation and immune activation capacity, as well as the unmature‐Tim4^neg^ KC phenotype. We have demonstrated spontaneous cholestatic damage in immune‐restricted C/EBPβ deficient mice with high serum levels of bile acids and increased expression of the cholnagiocyte marker CK19 in the livers. One possible explanation for this phenomena might relate to the increased numbers of myeloid DC which can cause intrahepatic bile duct injury via the IL‐17 axis, as was demonstrated in biliary atresia model in mice.^26^ Of note, C/EBPβ deficiency leads to overexpression of systemic IL‐6 and Castleman like syndrome^27^, ^28^ and the phenotype we describe here could represent an early phase of this pathology.

Whole body C/EBPβ KO mice where shown to be protected from hepatic lipid accumulation, liver inflammation and fibrosis.^16^, ^17^ Here we show that immune restricted C/EBPβ mice do not recapitulate this phenotype, indicating that C/EBPβ expression by nonimmune cells, such as hepatocyte and hepatic stellate cells are important in this pathology, in agreement with previous suggestions.^12^, ^17^ Of note, Satoh et al.^10^ described a phenotype protected from lung and liver fibrosis development, in the CDA‐HFD liver model, using C/EBPβ^−/−^ chimeric mice that were generated by fetal liver cells transfer into WT recipients. The findings were attributed to lack of a specific cell in C/EBPβ‐deficient chimeras, termed segregated‐nucleus‐containing atypical monocytes (SatM), that resemble Ly6C^low^ monocytes. The difference in the phenotype observed between this study and our study remains unknown and might be due to the use of fetal liver versus bone marrow cells for the transfer.

In conclusion, we have investigated the liver of immune restricted C/EBPβ‐deficient mice and found major alteration in the composition of the intra‐hepatic myeloid cells pool associated with mild spontaneous hepatitis in steady state. Yet, immune cell restricted C/EBPβ‐deficiency did not confer protection from hepatic steatosis, inflammation and fibrosis, that are suggested to be influenced by C/EBPβ expression in nonimmune cells.

## AUTHOR CONTRIBUTIONS

**Ayelet Kaminitz and Debby Reuveni**: performed the experiments. **Ayelet Kaminitz and Ehud Zigmond**: designed the research study. **Itay Moshkovits**: performed flow cytometry analysis. **Metsada Pasmanik‐Chor**: performed bioinformatics analysis. **Eli Brazowski**: performed histopathology aassessment. **Alexander Mildner and Achim Leutz**: contributed essential reagents or tools. **Itay Moshkovits and Ehud Zigmond**: wrote the paper.

## Data Availability

The data that support the findings of this study are available from the corresponding author upon reasonable request.
